# miR-16-5p Promotes Erythroid Maturation of Erythroleukemia Cells by Regulating Ribosome Biogenesis

**DOI:** 10.3390/ph14020137

**Published:** 2021-02-09

**Authors:** Christos I. Papagiannopoulos, Nikoleta F. Theodoroula, Ioannis S. Vizirianakis

**Affiliations:** 1 Laboratory of Pharmacology, School of Pharmacy, Aristotle University of Thessaloniki, GR-54124 Thessaloniki, Greece; papagiac@pharm.auth.gr (C.I.P.); theodorn@pharm.auth.gr (N.F.T.); 2 FunPATH (Functional Proteomics and Systems Biology Research Group at AUTH) Research Group, KEDEK—Aristotle University of Thessaloniki, Balkan Center, GR-57001 Thessaloniki, Greece; 3 Department of Life and Health Sciences, University of Nicosia, CY-1700 Nicosia, Cyprus

**Keywords:** miRNA therapeutics, miR-16-5p, erythroleukemia, erythroid differentiation, cancer, ribosomes

## Abstract

miRNAs constitute a class of non-coding RNA that act as powerful epigenetic regulators in animal and plant cells. In order to identify putative tumor-suppressor miRNAs we profiled the expression of various miRNAs during differentiation of erythroleukemia cells. RNA was purified before and after differentiation induction and subjected to quantitative RT-PCR. The majority of the miRNAs tested were found upregulated in differentiated cells with miR-16-5p showing the most significant increase. Functional studies using gain- and loss-of-function constructs proposed that miR-16-5p has a role in promoting the erythroid differentiation program of murine erythroleukemia (MEL) cells. In order to identify the underlying mechanism of action, we utilized bioinformatic in-silico platforms that incorporate predictions for the genes targeted by miR-16-5p. Interestingly, ribosome constituents, as well as ribosome biogenesis factors, were overrepresented among the miR-16-5p predicted gene targets. Accordingly, biochemical experiments showed that, indeed, miR-16-5p could modulate the levels of independent ribosomal proteins, and the overall ribosomal levels in cultured cells. In conclusion, miR-16-5p is identified as a differentiation-promoting agent in erythroleukemia cells, demonstrating antiproliferative activity, likely as a result of its ability to target the ribosomal machinery and restore any imbalanced activity imposed by the malignancy and the blockade of differentiation.

## 1. Introduction

MicroRNAs (miRNAs) have been implicated in all major biological processes that govern the metabolism and function of cells as well as the life of organisms. They regulate gene expression by base-pairing with complementary target mRNA molecules and typically induce RNA silencing. Frequently, miRNAs are found amplified or downregulated in cancer cells acting either as oncogenes (onco-miRs) or as tumor suppressor molecules [1,2,3,4,5]. For instance, miR-21 is overexpressed in a variety of cancer cell types and promotes carcinogenesis by repressing the levels of tumor-suppressor genes such as, PTEN, FasL and PDCD4 [6,7]. On the contrary, let-7 [8] and miR-34a [9] have shown tumor-suppressor properties, by inhibiting oncogenic cell signaling (via the RAS oncogene) and cell cycling (activation of TP53), respectively. The group of suppressor miRNAs comprises an interesting class of naturally occurring antineoplastic agents and many are in ongoing clinical trials for cancer therapy. Thus, the identification and characterization of novel oncosuppressor miRNAs for specific tumor types will advance the development of new anticancer therapeutic strategies. 

Erythroleukemia, a subtype of acute myeloid leukemia (AML), is a hyperproliferative disease caused by the multiplication of erythroid precursor cells and associates with poor prognosis. As with other cancer types, erythroleukemias show an imbalance in favor of hyperproliferative phenotypes over differentiation. However, extensive work in established cell line erythroleukemia models (especially murine erythroleukemia, MEL, cells) has shown that the leukemic phenotype can be reversed in vitro by reintroducing the cells into the path of differentiation [10,11,12,13,14,15,16]. Differentiation is initiated after treatment with chemical inducers (such as hexamethylene bisacetamide, HMBA) and involves an extensive yet precisely orchestrated gene expression program [10,11,17]. MiRNAs may play significant roles in this complex process of erythroid differentiation. For instance, miR-451 the best studied miRNA in erythropoiesis, undergo substantial overexpression and promotes erythroid differentiation by repressing the master-transcription factor GATA-2 [18,19,20]. On the contrary, miR-126 [21] and miR-15a [22] act as negative regulators of erythroid differentiation by repressing the levels of c-MYB. Similarly, a role for inhibiting differentiation has been assigned to miR-223 through interaction with LMO2 [23]. Thus, individual miRNAs act as crucial genetic regulators in differentiation, while those that promote the process might act as tumor-suppressor molecules of potential pharmacological interest.

In order to identify novel miRNAs that participate in the erythroid differentiation program, we analyzed the expression of 20 miRNAs using quantitative reverse transcription PCR (qRT-PCR) in MEL cells. By profiling the expression levels before and after the induction of differentiation, we generated a comprehensive expression map of miRNA levels and identified various compelling tumor-suppressor candidates. We focus on miR-16-5p, which showed a strong upregulation in differentiated MEL cells. Previously, miR-16-5p was shown to inhibit the proliferation rates of breast cancer cell lines [24,25,26]. Via the use of mimic and inhibitory miR-16-5p recombinant vectors we reproduce this data and, also, show that miR-16-5p was capable of promoting the differentiation program of MEL cells. Moreover, bioinformatic analysis of the miR-16-5p target genes revealed a plethora of ribosome biogenesis factors as putative genes affected by miR-16-5p. Indeed, miR-16-5p modulated the ribosomal function of MEL cells by downregulating specific ribosomal proteins and affecting the overall ribosomal levels in the cytoplasm. This effect may provide an explanation on the erythroid-related phenomena and the tumor-suppressor properties of miR-16-5p in cell lines of other origin. In conclusion, miR-16-5p is a potent tumor-suppressor molecule, which holds promise for pharmacological exploitation in erythroid-related disorders.

## 2. Results

### 2.1. A Comprehensive miRNA Gene Expression Screening Uncovers miR-16-5p as a Potential Regulator of the MEL Cell Differentiation Program

The MEL cell line represents an established in-vitro system to study fundamental mechanisms of erythroid differentiation and obtain valuable insights into red cell-related disorders [10,11,12,13,15,17]. While these cells are blocked at the proerythroblast stage of differentiation and are highly tumorigenic, they can be induced to differentiate by chemical compounds, such as HMBA, and become erythrocyte-like. Indeed, after treatment with HMBA (5 × 10^−3^ M), cells commit to differentiate and reproduce all the main hallmarks of erythroid differentiation as they (a) escape their malignant nature (Figure S1A,B), (b) gradually decrease in size (Figure S1E) and (c) accumulate vast amounts of hemoglobin both at mRNA and protein level (Figure S1C,D).

We decided to profile miRNA expression in the MEL system using RT-qPCR, due the high sensitivity and specificity of the assay over other methodologies, such as microarrays. The U6 small nucleolar RNA, a commonly used internal control gene in miRNA RT-qPCR assays, was employed as a reference molecule, due to its ubiquitously high expression levels. Interestingly, by comparing the independent Ct values of each miRNA, we observed that miR-16-5p was consistently the first miRNA to be amplified (lowest Ct) in three independent experiments (Figure S2A,B).

**Figure 1:**
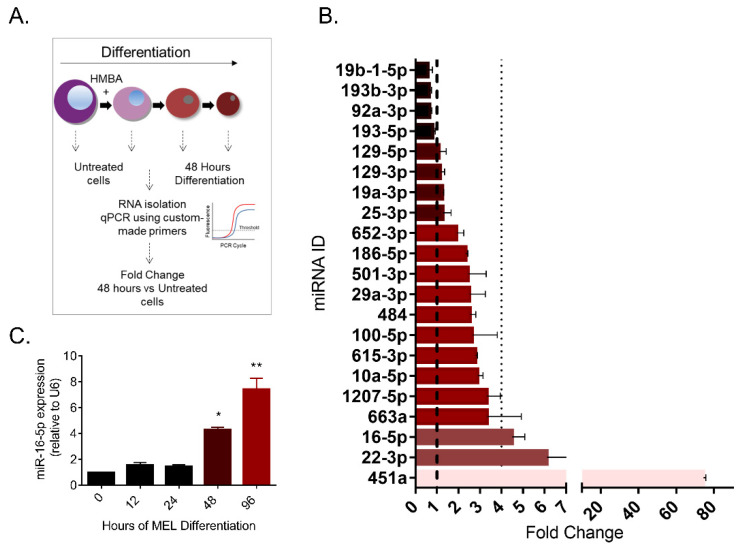
miRNA levels during murine erythroleukemia (MEL) cell differentiation. (**A**) Schematic representation of the miRNA screening performed in Figure 1. (**B**) Alterations in the levels of miRNAs in HMBA 48 h treated cells against untreated cells, ranked from the most upregulated miRNA to the most downregulated. All values were normalized to U6 expression. (**C**) MiR-16-5p cellular levels during MEL differentiation in a time-dependent manner. One-way analysis of variance performing multiple comparisons of each time point against 0 h (* *p* = 0.0005, ** *p*< 0.0001).

Moreover, we tested whether the miRNA expression levels vary between the untreated and differentiated MEL cells (48 h HMBA post-treatment, experimental design is outlined in Figure 1A). MiR-451, the well-characterized miRNA regulator of erythropoiesis, is known to undergo dramatic overexpression in differentiated cells, a feature that is present in our data analysis scheme and validates the methodology we used (Figure 1C) [18,19]. Additionally, most miRNAs were found overexpressed in this setting (16 out of 20), an observation that is consistent with previous studies showing a general miRNA increase during erythroid differentiation [27]. On the contrary, a small group of four miRNAs showed a reduction in their levels after differentiation was induced (miR-193-5p, miR-92a-3p, miR-193b-3p and miR-19b-1-5p, Figure 1B). Interestingly, of all the miRNAs tested, miR-16-5p and miR-22-3p were most significantly overexpressed (>4-fold) (Figure 1B), denoting that they might regulate critical aspects of the process of differentiation. Based on this data, miR-16-5p was selected for further analysis, as it: (a) showed high relative abundance in untreated cells (lowest Ct value, Figure S2) and (b) it was significantly overexpressed after the induction of erythroid differentiation of MEL cells (Figure 1B). Finally, miR-16-5p expression was profiled in a time- dependent experiment, which showed that overexpression is mildly initiated after 12 h but reaches significant levels after 48 h of HMBA treatment (Figure 1C).

### 2.2. miR-16-5p Overexpression Suppresses the Proliferation Potential of Various Cancer Cell Types

To study the miR-16-5p mediated phenotypes, we exogenously increased the miRNA levels by transfecting a panel of cell lines with a miR-16-5p containing recombinant plasmid (pcDNA3-pri-miR-16-1, denoted as mimic). To monitor transfection efficiency we performed parallel transfections with a GFP-reporter plasmid (Figure S4). Moreover, the levels of the induced miRNA overexpression were determined through qRT-PCR detection of miR-16-5p (Figure S4). In all four cell lines tested we achieved significant overexpression of miR-16-5p by this protocol (Figure S4). Additionally, to validate that the plasmid represents an appropriate tool for the study of miR-16-5p, we reproduced previously published data, which has shown that miR-16-5p overexpression suppresses the proliferation capacity of breast cancer cell lines [24]. Indeed, transfection with the miR-16-5p mimic plasmid significantly slowed the proliferation rates of the human MCF-7 and MDA-MB-231 breast cancer cell lines (Figure S5C,D), in comparison to cells mock-transfected cells with an empty vector (E.V., PCDNA3.1). We further expanded this spectrum of anticancer activity of miR-16-5p against the erythroleukemia MEL cells, and the human embryonic kidney 293 cells (HEK293), as shown in Figure S5A,B, respectively. Taken together, miR-16-5p demonstrates potent tumor-suppressor activity against different tumor histological types, as proposed by the proliferation behavior of all the four transfected cancer cell lines.

### 2.3. miR-16-5p Overexpression Increases Erythroid Differentiation of MEL Cells

**Figure 2:**
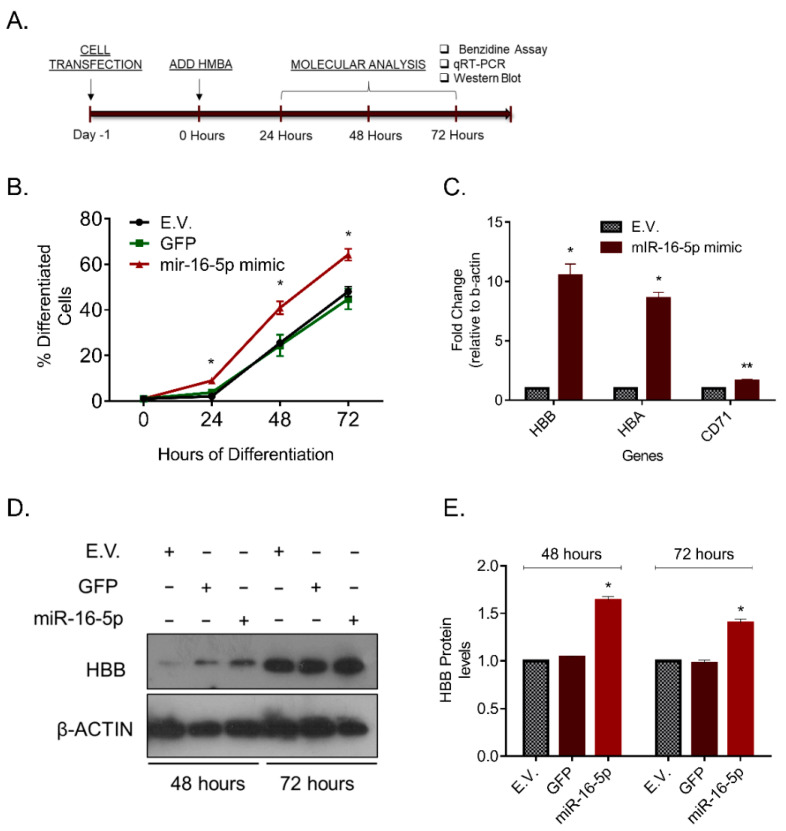
The exogenous overexpression of miR-16-5p increases erythroid differentiation. (**A**) Timeline depicting the cell treatments performed for the experiments in Figure 2. Transfection with miR-16-5p mimic was performed in day-1 and HMBA treatment was initiated in day-0. (**B**) Percentage of differentiated cells in a time-dependent experiment for the indicated cell cultures. Differentiation was scored using the benzidine positivity assay, which stains hemoglobin-positive cells. (**C**) mRNA levels of selected biomarkers of differentiation in E.V. against miR-16-5p mimic treated cells (normalized to β-actin). (**D**) Western blot using specific antibodies against hemoglobin-β (HBB) and β-actin in protein lysates derived from E.V., GFP and miR-16-5p mimic (48- and 72-h treatment). (**E**) Quantification of the Western blot analysis in D. Statistical significance was inferred using multiple *t*-tests (Holm–Sidak method) between E.V. and miR-16-5p mimic treated cells (* *p* < 0.001, ** *p* < 0.0001).

Since miR-16-5p is strongly upregulated during the late stages of MEL cell differentiation, we hypothesized that the exogenous overexpression of this miRNA would increase the accumulation of terminally differentiated cells in culture. To test this hypothesis, miR-16-5p levels were elevated by employing the methodology of the plasmid transfection (pcDNA3-pri-miR-16-1), before inducing cells to differentiate with HMBA, as outlined in Figure 2A. Importantly, we found that the elevated miR-16-5p levels forced an superior number of cells to reach terminal erythroid maturation compared to control cells, as evidently shown by the colorimetric benzidine staining, a dye that binds directly to hemoglobin in living cells (Figure 2B). Similarly, the assessment of established erythropoiesis markers have indicated increased expression of hemoglobin-β, hemoglobin-α and CD71 (transferrin receptor) by qRT-PCR analysis (Figure 2C), and the hemoglobin-β protein by Western blot analysis (Figure 2D,E) in miR-16-5p transfected cells. Thus, the overexpression of a single miRNA is capable of increasing the number of MEL cells that reach terminal differentiation.

**Figure 3:**
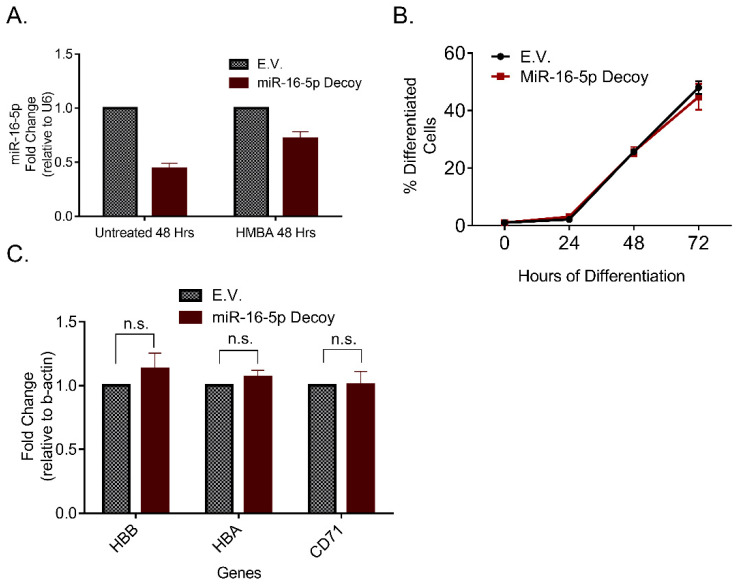
Inhibition of miR-16-5p by a decoy plasmid in MEL cells. (**A**) Quantification of the miR-16-5p cellular levels after transfection with the decoy plasmid. The experiment was performed exactly as with the mimic plasmid shown in the timeline in Figure 2A, thus, transfection with the decoy plasmid was performed in day-1. (**B**) Percentage of differentiated cells in each culture in a time-dependent experiment. Differentiation was scored using the benzidine positivity assay, which stains hemoglobin-accumulating cells. (**C**) mRNA levels of selected biomarkers of differentiation in E.V. against miR-16-5p decoy treated cells (normalized to β-actin). Statistical analysis was performed using multiple *t*-tests (Holm–Sidak method) between E.V. and miR-16-5p decoy treated cells and no alteration was found to be statistically significant.

Having established a correlation between miR-16-5p overexpression and enhanced erythroid differentiation, we also reasoned to search at the opposite direction that of miRNA inhibition. Decoy plasmids, which contain hundreds of miRNA target sites, are used to sponge a miRNA, triggering its subsequent degradation. By transfecting MEL cells with such a miR-16-5p decoy plasmid (AB.pCCL.sin.cPPT.U6.miR-16-Decoy.hPGK.GFP.WPRE), we were able to reduce miR-16-5p levels in untreated (uninduced to differentiate) cell cultures and, also, to prevent the described above upregulation of the miR-16-5p during the execution of the erythroid differentiation program (Figure 3A). However, when we triggered differentiation with HMBA, we observed that the decoy treated cells had differentiation rates similar to E.V. treated cells (Figure 3B). This was seen both in the colorimetric benzidine assay (hemoglobin-producing cells, Figure 3B) and by qRT-PCR analysis of the mRNA levels of hemoglobin-β, hemoglobin-α and CD71 (Figure 3C). Thus, while the miR-16-5p exogenous upregulation increased the differentiation capacity, its corresponding inhibition did not hinder erythropoiesis, likely due to additional miRNAs compensating for the loss of function of miR-16-5p.

### 2.4. Bioinformatic Analysis Reveals Ribosome Constituents as the Main miR-16-5p Target Genes

**Figure 4:**
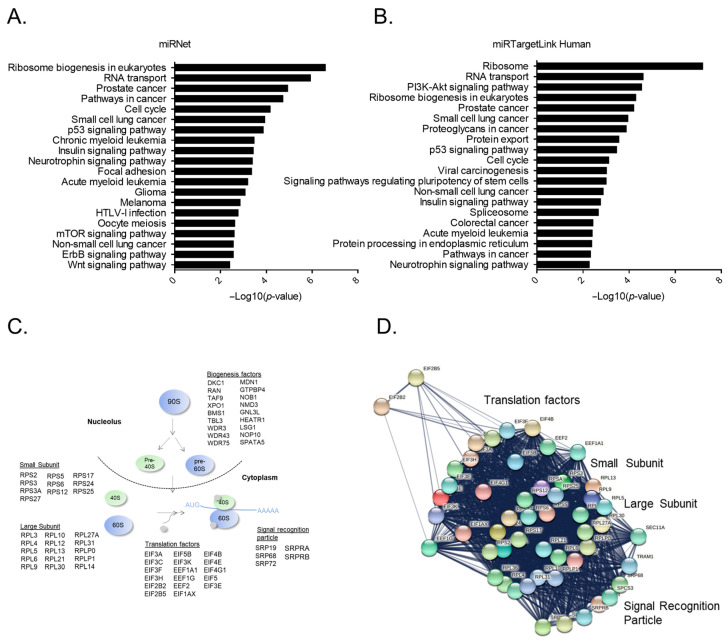
Bioinformatic analysis of the miR-16-5p target genes. (**A**,**B**) Functional enrichment analysis of the most significant pathways among the miR-16-5p target genes retrieved through (**A**) miRNet and (**B**) miRTargetLink. Each bar plot represents a KEGG pathway ranked in a descending order based on their calculated *p*-values. The analysis was performed using the miRNET functional enrichment tool. (**C**) Graphical representation of major steps of ribosome biogenesis, translation initiation and regulation, depicting the genes affected by miR-16-5p. (**D**) Protein–protein interaction network between the genes shown in B. The network analysis was performed with the String database tool. Network edges represent protein–protein interactions, sourced from text mining, experiments, databases, coexpression, neighborhood, gene fusion and co-occurrence.

To obtain insights into the underlying miR-16-5p mechanism of action, we performed a bioinformatic data search concerning the potential target-genes bound by miR-16-5p. To this end, we utilized three bioinformatic platforms: miRNET [28], miRTargetLink [29] and miRWalk [30] that incorporate miRNA-target gene data from various well annotated databases. Interestingly, the data from miRNET and miRTargetLink were highly correlated as 1384 miR-16-5p target genes appear in both databases (Figure S6). However, there was only mild correlation of those two databases with miRWalk, likely due to the fact that miRWalk incorporates predicted interactions, whereas miRNET and miRTargetLink mainly focus on validated interactions. To extract functional insights out of the validated interactions retrieved through miRNET and miRTargetLink, we performed pathway analysis and found that genes related to the ribosomal machinery comprise the most significant target group of miR-16-5p (Figure 4A,B, and Supplementary Materials excel file). Interestingly, this group of genes contains factors that participate in major steps of both ribosome biogenesis in the nucleolus, but also ribosome assembly and translation in the cytoplasm (Figure 4C). Moreover, by building a protein–protein interaction network, we found that the ribosome related factors formed a dense cluster with great connectivity between the associated nodes (Figure 4D), denoting that these proteins are likely coregulated. In conclusion, the bioinformatic findings suggest that miR-16-5p may modify ribosomal function within eukaryotic cells.

### 2.5. miR-16-5p Reduces the Overall Ribosomal Levels in MEL Cells

**Figure 5:**
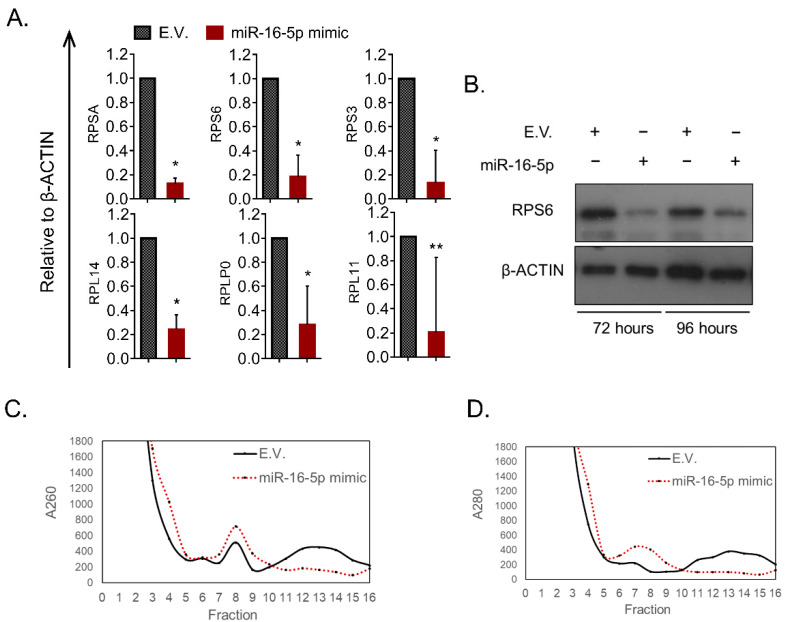
miR-16-5p overexpression modulates ribosomes in MEL cells. (**A**) mRNA levels of selected members of the small and large subunit of the ribosome in E.V. against miR-16-5p mimic treated cells. All genes were normalized to β-actin expression. Statistical significance was inferred using a two-tailed *t*-test between E.V. and miR-16-5p mimic treated cells. * *p* < 0.001, ** *p* < 0.05. (**B**) Protein levels of RPS6 after 72 and 96 h of treatment with either E.V. or miR-16-5p mimic plasmid. (**C**) Ribosome fractionation through ultracentrifugation in a sucrose gradient, derived from cells treated with E.V. or miR-16-5p. After the ultracentrifugation the gradient was separated into 16 fractions and absorbance at 260 nm was determined using a spectrophotometer. Fractions 7-8 correspond to monosomal particles and fractions 10–16 are mainly populated by polysomes. (**D**) Same as with C but here absorbance at 280 nm was determined.

In order to study the potential relationship between ribosomes and miR-16-5p, the mimic plasmid (pcDNA3-pri-miR-16-1) was re-employed. Cells transfected with the mimic recombinant plasmid and the E.V. construct was used to isolate their total cytoplasmic RNA and assess the levels of selected miR-16-5p-target genes. Interestingly, the mRNA levels of representative factors of the small (RPSA, RPS6 and RPS3) and the large (RPL14, RPLP0 and RPL11) ribosomal subunits were found consistently reduced after miR-16-5p overexpression (Figure 5A). Similarly, the RPS6 protein levels assessed by Western blotting were, also, significantly lowered in cells transfected with the miRNA mimic recombinant plasmid (Figure 5B).

The extensive list of ribosomal factors targeted by miR-16-5p and the high connectivity between them, led us to hypothesize that miR-16-5p may affect the overall ribosome levels in MEL cells. To test this hypothesis, we fractionated ribosomes, derived from E.V. and miR-16-5p mimic-transfected MEL cells, inside a 10–50% sucrose gradient. Using this assay, the individual ribosomal subtypes (free subunits, monosomes and polysomes) are forced to separate based on their density by applying extreme force through ultracentrifugation. The absorbance values of 260 nm (A260) and 280 nm (A280) provide a good indicator of the levels of the corresponding ribosomal RNA and ribosomal proteins at each part of the gradient, respectively [31]. Notably, we found that miR-16-5p was capable of reducing the overall levels of polysomes, which comprise the most translationally active and numerous subtype of ribosomes (Fractions 10-16 in Figure 5C,D). On the contrary, the levels of the poorly active monosomal particles were found slightly increased in miR-16-5p cells (Fractions 6–8 in Figure 5C,D). These findings are depicted in both A260, representing ribosomal RNA abundancy, and A280 values, corresponding to the proteinaceous compartment of the ribosome. Taken together, miR-16-5p alters ribosomal function in MEL cells by inducing an extensive reprogramming of the translational machinery towards less active ribosomal subtypes.

## 3. Discussion

MicroRNAs are involved in almost every aspect of cancer cell biology acting either as oncogenes or as tumor-suppressors. In this work, it was hypothesized that certain miRNA candidates may be pharmacologically exploited and used therapeutically by inducing differentiation of erythroleukemia cells. To study this association, we performed a miRNA expression screening in MEL cells and identified various putative regulators of the erythroid differentiation program.

Interestingly, we observed that the majority of the miRNAs tested underwent upregulation during late differentiation. On the contrary, only four miRNAs were, albeit mildly, downregulated. Similar conclusions were reached by Zhan et al. by also showing a general miRNA increase during differentiation [27]. Given that a universal reduction in protein abundancy is a hallmark of erythroid differentiation, this group of upregulated miRNAs may complement other pathways (such as proteasomal degradation and autophagy) in order to induce a widespread quantitative proteome decline [32]. Each miRNA may target hundreds of unique or redundant genes; thus the cumulative effect of multiple miRNAs is of vast buffering capacity. In conclusion, a general increase in miRNA expression likely represents a major event of erythroleukemia differentiation and likely plays a role in proteome reorganization.

Moreover, miR-16-5p is identified, in this study, as a tumor-suppressor in MEL cells. Additional studies in varying cancer cell types have, also, assigned a similar role for miR-16-5p. For instance, miR-16-5p is frequently deleted in patients with chronic lymphocytic leukemia [33,34,35] and downregulated in breast cancer patient samples [24,25]. In these cell types, miR-16-5p was shown to exert activity through binding to BCL-2 and VEGFA, respectively. MiR-16-5p was, also, implicated with replication of enterovirus 71 (EV71) [35], and had a role related to drug response in malignant mesothelioma [36]. We, thereby, complement this repertoire of miR-16-5p activity, by showing that miR-16-5p not only reduced the proliferation potential of the malignant MEL cells, but also increased terminal erythroid differentiation. Thus, expression of miR-16-5p is an excellent target for pharmacological intervention against a variety of malignancies.

Highlighted in this work, ribosome targeting is a novel function of miR-16-5p that may explain the tumor-suppressor properties of this miRNA. This effect was found through searching in two independent databases, which have, previously, provided critical resources for the identification of actionable miRNAs in various types of cancer and in diet and in physical activity [37,38,39,40,41]. Moreover, studies over the past years have shown that ribosomes are heavily modified in malignancies in order to meet the increment metabolic needs of the cancer cell. For this reason, ribosome biogenesis becomes overactivated and supports growth by vastly increasing ribosome supplies available for translation in the cytoplasm [42]. On the contrary, cancer cells, which are forced to reduce their ribosomes, become vulnerable to cell death [43]. The data in this study suggests that miR-16-5p targets a plethora of factors that functionally belong to ribosome biogenesis and assembly, ribosome structure and regulation. Thus, miR-16-5p may reverse the quantitative alterations of the ribosome by targeting both ribosome biogenesis and active ribosomes in the cytoplasm. Notably, it appears to target the polysomal fraction of ribosomes, which represent the most actively engaged in translation particles and account for vast majority of the translation activity inside a cell. Additionally, the slight increase of monosomal fractions may represent intermediate ribosomal subtypes, which are destined for destruction, as polysomes must first become monosomes and then be degraded. Finally, it is tempting to hypothesize that ribosome alterations may also account for the effects seen by miR-16-5p in other cancer cell types (such as in breast cancer).

The ribosome related effects of miR-16-5p might also explain the pro-differentiation properties seen in MEL cells. Studies over the past years have shown that there is stringent and precise regulation of ribosome levels and function during erythropoiesis. This is evident under conditions of mild ribosome dysfunction, as it occurs in a group of disorders termed ribosomopathies [14,44,45]. The latter are caused by heterozygous mutations in ribosomal proteins and associate with highly specific symptoms. In particular, patients demonstrate a notable inability to produce red blood cells with very limited toxicity in other tissues, despite the ubiquitous necessity of ribosomes for all cell types [46]. Moreover, previous research from our group has shown that ribosome regulation mediated by the cell-fate transcription factors PU.1 and GATA-1 is an integral part of erythroleukemia differentiation [47]. This ribosome reorganization occurs during the early stages of differentiation (12 h of treatment) and represents a necessary step for erythropoiesis progression [47,48]. Thus, miR-16-5p likely exerts its pro-differentiation effects by promoting ribosome downregulation during the early or intermediate steps of the differentiation process to erythroid maturation. To this notion concurs the data that miR-16-5p reprograms ribosomes, by reducing polysomes and increasing monosomes, overall, towards less active ribosomal subtypes. Likely, by inducing these alterations, cells are more prepared for completing additional hallmarks of differentiation, such as cell cycle exit, limitation of the proliferation rate, or chromatin condensation [10,11,17].

A potential limitation of our study is the use of a single, murine, model of erythroid differentiation. The accumulated biological knowledge on the MEL cell system has shown that while it represents a great model of erythroid differentiation (Figure S1) it does not fully recapitulate all aspects of human normal erythropoiesis. For instance, the untreated MEL cells are proerythroblast like cells, whereas the fully differentiated cells mirror orthochromatic normoblasts. As a consequence, the differentiation of MEL cells represents a part of human normal erythropoiesis and not the whole process. Nevertheless, major mechanisms of erythroid differentiation, including the role of critical transcription factors and erythroid specific miRNAs, have been identified in MEL cells [10,11,17]. Thus, we suggest that a validation of our findings in clinical samples and primary cells would significantly reinforce the potential pharmacological exploitation of miR-16-5p in erythroleukemia and other erythroid related disorders.

Nevertheless, the fact that a single miRNA, miR-16-5p, promotes erythroid differentiation of a cell line model is unexpected and calls for further investigation in the direction of its potential clinical utilization. MiRNA-based therapeutics has been shown to have numerous benefits, such as the ability to target multiple genes, with a critical role in cancer development and progression. The disadvantages, however, are mainly related to challenges in delivery, stability, off-target effects and safety [49,50]. To overcome these limitations the development of delivery platforms that allows maximum stability of the therapy and provide sufficient uptake capacity in target cells, while minimizing off-target side effects, is necessary. In this direction we previously employed exosomes derived from human MRC-5 cells and efficiently loaded them with siRNA/miRNA cargos [51]. Exosomes were efficient “bio-shuttles” by delivering miRNA molecules in a variety of cancer cell types. Thus, a future direction for miR-16-5p research would be a potential encapsulation into exosomes and delivery of the complexes into erythroleukemia animal models and in the long term the execution of clinical studies for market approval and delivery to humans.

## 4. Materials and Methods

### 4.1. MEL Cell Culture and Induction of Differentiation

The established permanent cancer model MEL-745 (murine erythroleukemia FLC clone 745) were handled in a way to maintain their passages with high inducibility of erythroid differentiation. To this end, cells were diluted every 3 days with fresh medium and maintained at a concentration between 5 × 10^4^ and 5 × 10^5^ cells/mL. The MEL cells were obtained from Dr. C. Friend (Division of Cytology, The Sloan-Kettering Institute for Cancer Research, New York, NY, USA) and they were grown in an incubator under standard conditions. Erythroid differentiation was induced after in culture treatment with 5 × 10^−3^ M HMBA dissolved in water.

### 4.2. RNA Extraction and Reverse Transcription-Quantitative Polymerase Chain Reaction (RT-qPCR) for miRNA Analysis

At least 10^6^ cells were harvested for RNA isolation with the miRNeasy Mini Kit (Qiagen Inc., Chatsworth, CA, USA), which enables enrichment of small RNAs. The extracted RNA was assessed for purity and integrity by two methods: (1) the use of a nanodrop 2000 spectrophotometer and (2) through the electrophoresis on an agarose gel and visualization of the integrity of the main ribosomal RNAs (28S and 18S). CDNA synthesis was performed with miScript II RT Kit (Qiagen Inc., Chatsworth, CA, USA). The latter attaches a universal tag at the 3′ end of each miRNA, thus increasing the length of each miRNA, in order to facilitate binding of two primers of 20 nucleotide length. The real time PCR analysis was performed with the help of miScript SYBR^®^ Green PCR Kit using a forward primer (custom-made) and a universal (common for all miRNAs) reverse primer. The latter binds to the universal tag attached to all miRNAs, and it is supplied by the manufacturer. The primers were constructed as described in the study by Hyejin Yoon et al. [52] and their sequences are shown in Table S3. QRT-PCR was performed in an Applied Biosystems^®^ 7500 fast instrument (ThermoFisher Scientific, Waltham, MA, USA). According to the manufacturer’s instructions the end product of the PCR should be approximately 80-100 bases, which is, indeed, the length of the all the miRNA we quantified (Figure S3). The analysis of the qRT-PCR data was performed using the 2−ΔΔCT method by normalizing to U6 expression.

The 20 miRNAs that we included in our analysis have been selected based on their previous reported or suspected involvement in aspects of erythroid differentiation. Moreover, to increase the number of miRNA candidates, we also selected some miRNAs with potential implications in the megakaryocyte-erythroid switch, as erythrocytes and platelets share a close common progenitor.

### 4.3. Cell Transfection

Transfection was performed with Lipofectamine 2000 (ThermoFisher Scientific, Waltham, MA, USA) as per the manufacturer’s instructions. The mimic (Catalog Number: 51382) and inhibitory plasmids (Catalog Number: 46593) were purchased from Addgene (Watertown, MA, USA). The eGFP plasmid was kindly provided by Dr. Eleni Nikolakaki (Laboratory of Biochemistry, Department of Chemistry, Aristotle University of Thessaloniki, Greece) and the empty pcDNA 3.1 vector was obtained from Invitrogen (Waltham, MA, USA). Optimization experiments were performed to find the optimal conditions for miRNA expression, and these were finally set to 1:2 DNA (in μg):Lipofectamine 2000 (in μL).

### 4.4. Cell Proliferation and Differentiation Assays

Cells proliferation was determined with the aid of the optical microscope in a Neubauer counting chamber (Paul Marienfeld GmbH & Co.KG, Lauda-Königshofen, Germany). Cell death was assessed using the Trypan blue dye-exclusion method, essentially as previously described [13]. Moreover, one of the methods used to score differentiation was the benzidine–H_2_O_2_ assay, which is performed directly in cultured cells. The assay was performed as previously described in [45], by assessing at least 300 cells of each culture.

### 4.5. Western Blot Analysis

Approximately 10^6^ cells of each cell culture were harvested, by centrifugation at 300× *g* for 5 min, and processed for total protein isolation and Western blot analysis. Pelleted cells were washed with PBS and lysed in lysis buffer (10 mM Tris-Cl pH 8.0, 150 mM NaCl, 2% SDS). The lysate was highly viscous due to DNA release and, therefore, it was sonicated for 30 s. The samples were then processed for protein quantitation using the BCA assay kit (ThermoFisher Scientific, Waltham, MA, USA). After protein quantification, one volume of protein sample was mixed with equal volume of Laemmli sample buffer (4% SDS, 20% glycerol, 10% 2-mercaptoethanol, 0.004% bromophenol blue and 0.125 M Tris HCl, pH 6.8) and heated for 5 min at 95 °C. Twenty micrograms of total protein material was loaded on a denaturing 12% SDS-PAGE gel and run until separation. Proteins were, then, transferred to a PVDF membrane and blotted with primary antibodies overnight at 4 °C and with secondary antibody for 1 h at room temperature. The antibodies used were from Santa Cruz Biotechnology (Dallas, TX, USA): Hemoglobin β/γ/δ/ε (sc-390668, diluted 1:200), β-actin (sc-47778, diluted 1:500), Rps6 (sc-74459, diluted 1:500) and m-IgGκBP-HRP (sc-516102, diluted 1:5000).

### 4.6. Bioinformatic Analysis of miR-16-5p Target Genes

The miR-16-5p target genes were retrieved from the miRNet [28], miRTargetLink [29] and miRWalk [30] online tools. The analysis returned 1557, 1606 and 617 target genes, respectively. Functional annotation of the miRNet targets was conducted with the embedded in miRNet annotation tool, using the following parameters: query was set to all genes, algorithm to the hypergeometric test and database to KEGG. Annotation of the miRTargetLink targets was performed with the DAVID bioinformatic tool [53,54]. The protein–protein interaction network analysis was performed with the string database tool [55,56,57]. Network edges represent protein-protein interactions, sourced from text mining, experiments, databases, coexpression, neighborhood, gene fusion and co-occurrence. Minimum required interaction score was set to medium confidence 0.400.

### 4.7. Ribosome Fractionation Protocol

For ribosome isolation, cells that grow with an exponential rate were treated, in culture, for 5 min with 100 μg/mL cycloheximide (Sigma-Aldrich, St. Louis, Missouri, United States), a translation elongation inhibitor that freezes and stabilizes the ribosome- mRNA-nascent peptide complex. Subsequently, 2 × 10^7^ cells were harvested and washed twice with PBS supplemented with 100 μg/mL cycloheximide. Cell lysis was achieved by exposure to mild detergent conditions after incubation with the following lysis buffer: 20 mM Tris pH 7.5, 150 mM NaCl, 15 mM MgCl2, 100 μg/mL cycloheximide, 1 mM DTT, 1%, Triton X-100, 6% glycerol, DNase I (AMPD1, Sigma-Aldrich, St. Louis, MO, USA), protease and phosphatase inhibitor (MSSAFE, Sigma-Aldrich, St. Louis, MO, USA). To ensure efficient cell lysis, samples were incubated on ice for 30 min. After cell lysis the samples were subjected to two rounds of centrifugation: a) for 5 min at 1800× *g*, 4 °C to pellet nuclei and b) 10,000× *g* for 5 min at 4 °C to pellet mitochondria. After the final centrifugation the supernatant was collected, and a portion was used to determine the light absorption at 260 nm (A260) at a spectrophotometer. At this point A260 corresponds linearly with the levels of ribosomes present at the lysate, due to the strong absorption of the four ribosomal RNAs, and thereby the value can be used to normalize between samples. We also determined protein concentration by BCA assay. Equal amounts of ribosomes were loaded on a 10–50% sucrose gradient. Samples were ultracentrifuged at 30,000 rpm for 3 h, using the low acceleration and brake options. After centrifugation the gradient was split into fractions of 0.75 mL with the help of a pipette. Finally, A260 was determined in each fraction as a measurement of ribosome abundancy at the corresponding part of the gradient.

## Data Availability

The data presented in this study are available on request from the corresponding author.

## Supplementary Materials

The following are available online at https://www.mdpi.com/1424-8247/14/2/137/s1, Figure S1: MEL cells as a model of erythroleukemia differentiation. Figure S2: miRNA relative levels in untreated MEL cells. Figure S3: Electrophoresis of the amplified products generated during the qRT-PCR experiment in Figure 1. Figure S4: Analysis of the transfection efficiency of GFP and miR-16-5p mimic plasmids. Figure S5: miR-16-5p overexpression inhibits cell proliferation of various cancer cell lines. Figure S6: Venn diagram comparing the genes targeted by miR-16-5p according to three online platforms (miRTargetLink, miRNet, miRwalk). Table S1: The 20 most significant pathways affected by miR-16-5p retrieved through miRNet (miRNet functional enrichment tool). Table S2: The 20 most significant pathways affected by miR-16-5p retrieved through miRTargetLink. Table S3: miRNA and mRNA primers used for the qRT-PCR assays in the study. Supplementary Excel File: Analysis of miR-16-5p target genes.
