# Supplementary material for: miR-16-5p Promotes Erythroid Maturation of Erythroleukemia Cells by Regulating Ribosome Biogenesis
Source: Pharmaceuticals (Basel). 2021 Feb 9;14(2):137. doi: 10.3390/ph14020137 (PMC7915806; doi:10.3390/ph14020137)
Supplement: Supplementary file 1 [file pharmaceuticals-14-00137-s001.zip › Supplementary material_Final.pdf]

pharmaceuticals

miR-16-5p Promotes Erythroid Maturation of Erythroleukemia Cells by Regulating Ribosome Biogenesis

Christos I. Papagiannopoulos [1], Nikoleta F. Theodoroula [1] and Ioannis S. Vizirianakis [1,2,3,*]

[1] Laboratory of Pharmacology, School of Pharmacy, Aristotle University of Thessaloniki, GR-54124 Thessaloniki, Greece; papagiac@pharm.auth.gr (C.I.P.); theodorn@pharm.auth.gr (N.F.T.)
[2] FunPATH (Functional Proteomics and Systems Biology Research Group at AUTH) Research Group, KEDEK—Aristotle University of Thessaloniki, Balkan Center, GR-57001, Thessaloniki, Greece
[3] Department of Life and Health Sciences, University of Nicosia, CY-1700 Nicosia, Cyprus
Correspondence: ivizir@pharm.auth.gr

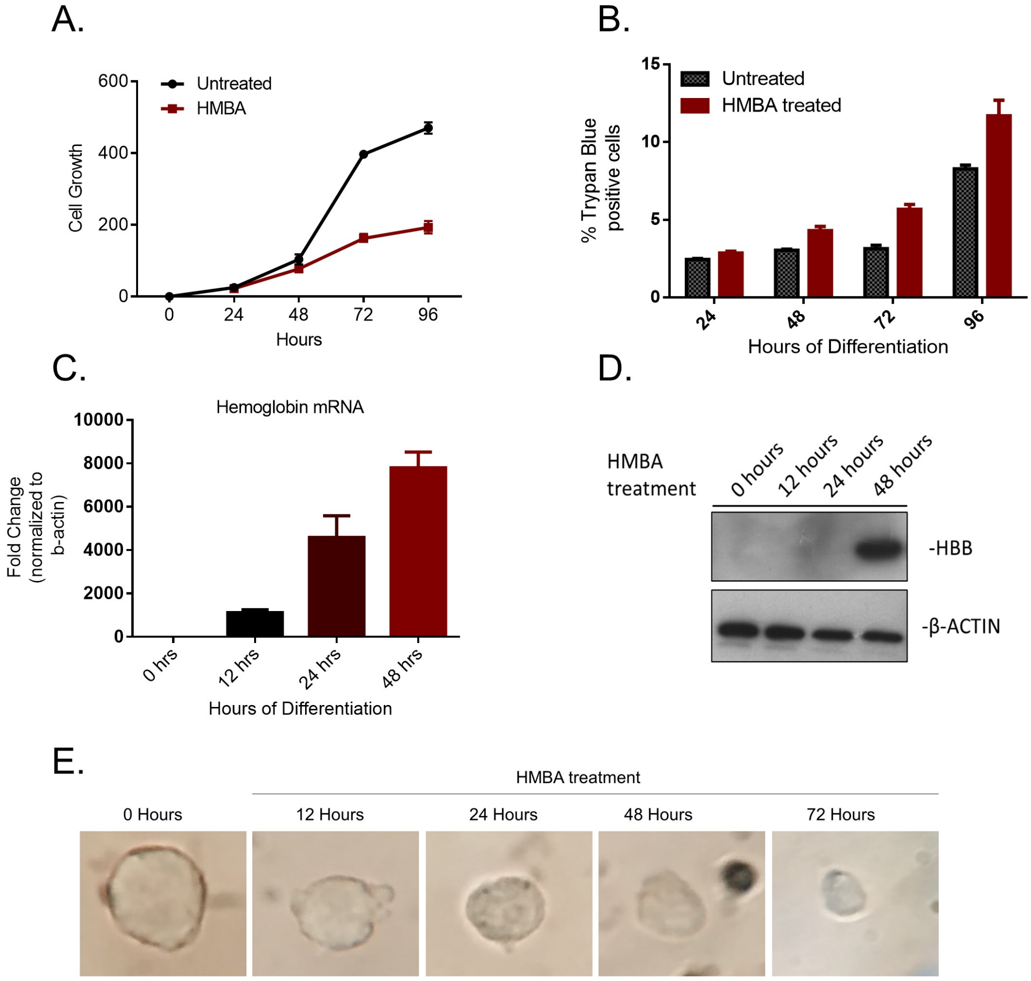

Figure S1. MEL cells as a model of erythroleukemia differentiation. (A) Kinetics of cell proliferation of MEL cells treated with HMBA (5 mM) in comparison to control untreated cultures. (B) Cell death in untreated against treated with HMBA cells for the indicated time-points, scored by trypan blue exclusion assay. (C) mRNA levels of hemoglobin-β (normalized to β-actin) after induction of differentiation with HMBA for the indicated time-points. (D) Hemoglobin-β protein levels after induction of differentiation with HMBA. (E) Differentiation-dependent decrease in cell size of MEL cultures observed under the optical microscope at a 100x magnification

A.

	miRNA ID	Ct Experiment 1	Ct Experiment 2	Ct Experiment 3	**Average Ct**	**SD**
Untreated MEL cells	U6	21,78	20,07	19,89	**20,58**	**0,85**
	16-5p	22,29	21,80	19,61	**21,23**	**1,16**
	92a-3p	22,61	20,91	20,93	**21,48**	**0,80**
	19a-3p	19,91	22,96	23,81	**22,22**	**1,68**
	25-3p	24,92	22,91	22,89	**23,57**	**0,95**
	29a-3p	26,91	24,93	24,91	**25,58**	**0,94**
	451a	26,07	26,91	26,79	**26,59**	**0,37**
	663a	26,59	32,50	20,94	**26,68**	**4,72**
	652-3p	27,92	25,92	27,81	**27,22**	**0,92**
	484	28,40	27,12	27,91	**27,81**	**0,53**
	22-3p	27,82	28,62	27,71	**28,05**	**0,40**
	193b-3p	30,49	28,89	25,67	**28,35**	**2,00**
	186-5p	27,91	28,42	28,87	**28,40**	**0,39**
	501-3p	31,94	28,92	29,17	**30,01**	**1,37**
	1207-5p	30,91	29,81	29,94	**30,22**	**0,49**
	129-5p	32,64	29,90	29,57	**30,70**	**1,37**
	19b-1-5p	30,82	29,94	31,69	**30,82**	**0,71**
	328-3p	32,68	30,91	31,18	**31,59**	**0,78**
	615-3p	32,91	31,95	31,94	**32,27**	**0,46**
	100-5p	33,91	31,90	31,90	**32,57**	**0,95**
	193-5p	34,90	32,53	32,93	**33,45**	**1,04**
	10a-5p	34,93	32,92	32,92	**33,59**	**0,95**

B.

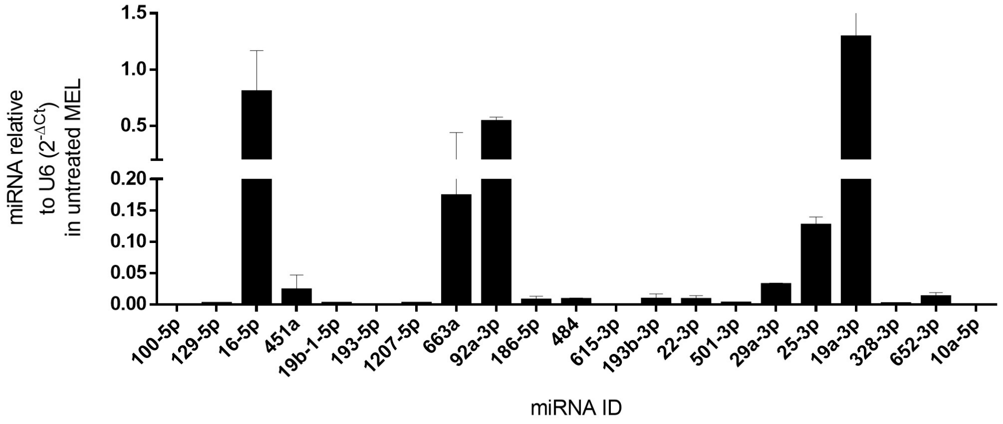

Figure S2. miRNA relative levels in untreated MEL cells. (A) Cycle threshold (Ct) values of all miRNAs quantified in three independent experiments. The values shown were generated in untreated (not induced to differentiate) MEL cells. (B) miRNA relative levels in untreated MEL cells using the average values shown in A. Each independent Ct value was normalized to U6 to calculate a ΔCt value. The $2^{-\Delta Ct}$ value for each miRNA is shown in the bar plot.

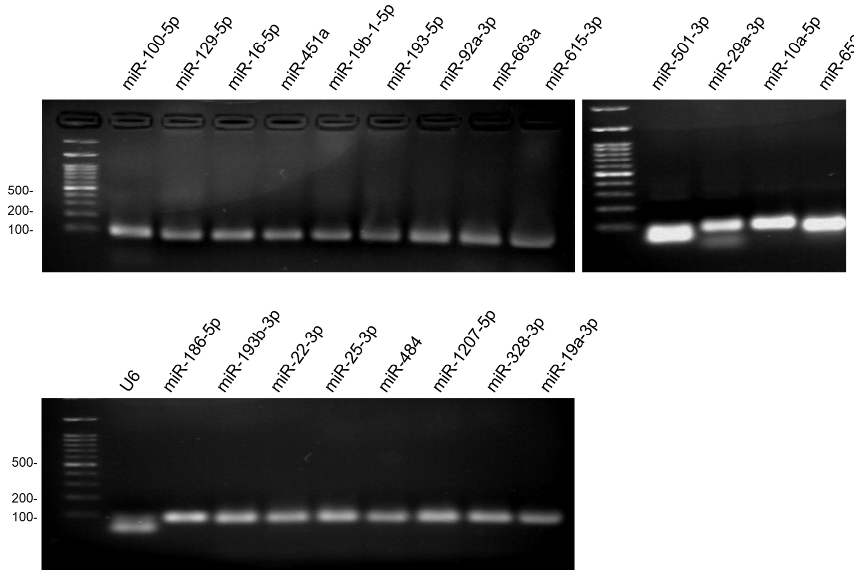

Figure S3. Electrophoresis of the amplified products generated during the qRT-PCR experiment in figure 1. Each miRNA with a size of 20-22 nucleotides is enriched with a universal tag of 60 nucleotides during reverse transcription. Thus, the final size of the quantified miRNAs should be ~80 nucleotides. Electrophoresis was performed inside a 2% agarose gel and fragments were stained with ethidium bromide.

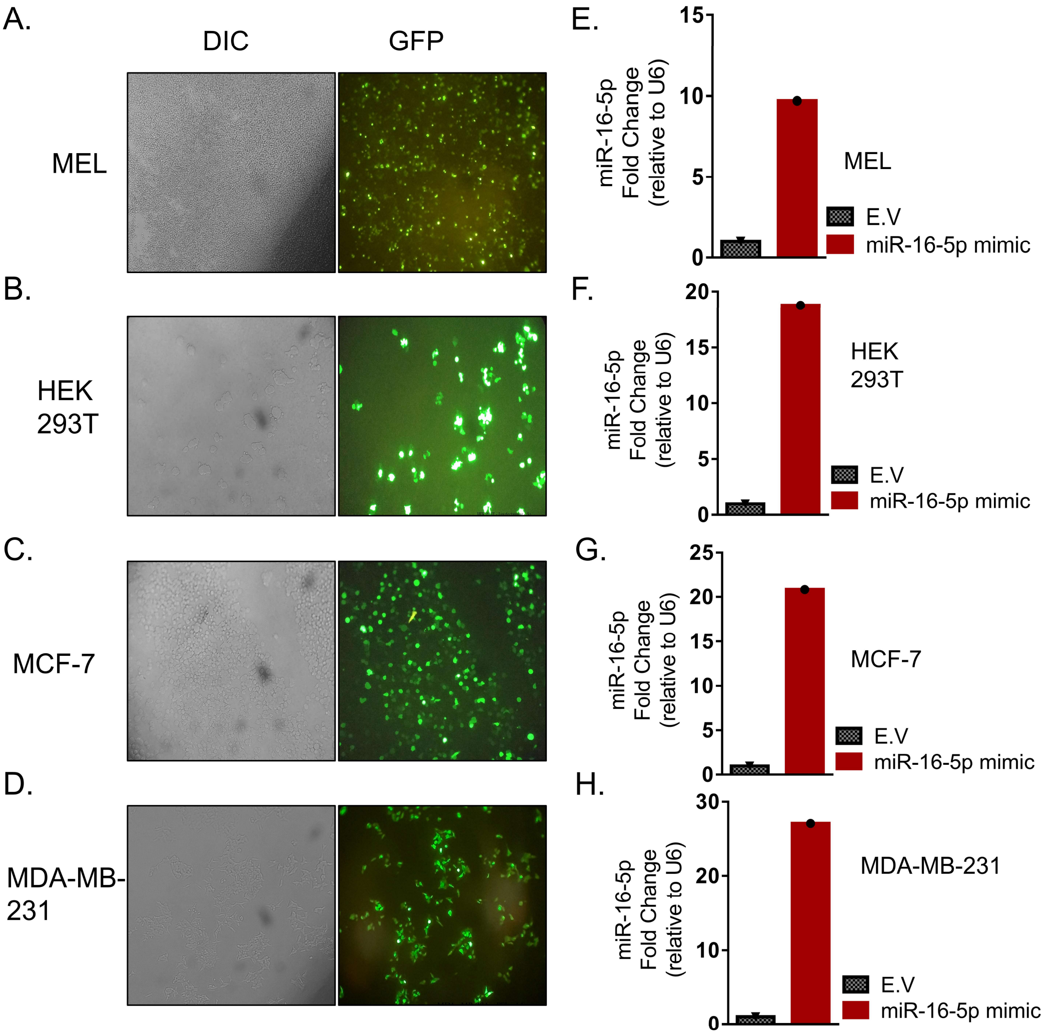

Figure S4. Analysis of the transfection efficiency of GFP and miR-16-5p mimic plasmids. (A-D) GFP was transfected into the indicated cell lines for 48 hours and fluorescence was assessed using a fluorescent microscope. (E-H) The indicated cell lines were transfected with miR-16-5p mimic for 48 hours and the fold change in the levels of mature miR-16-5p were scored by qRT-PCR.

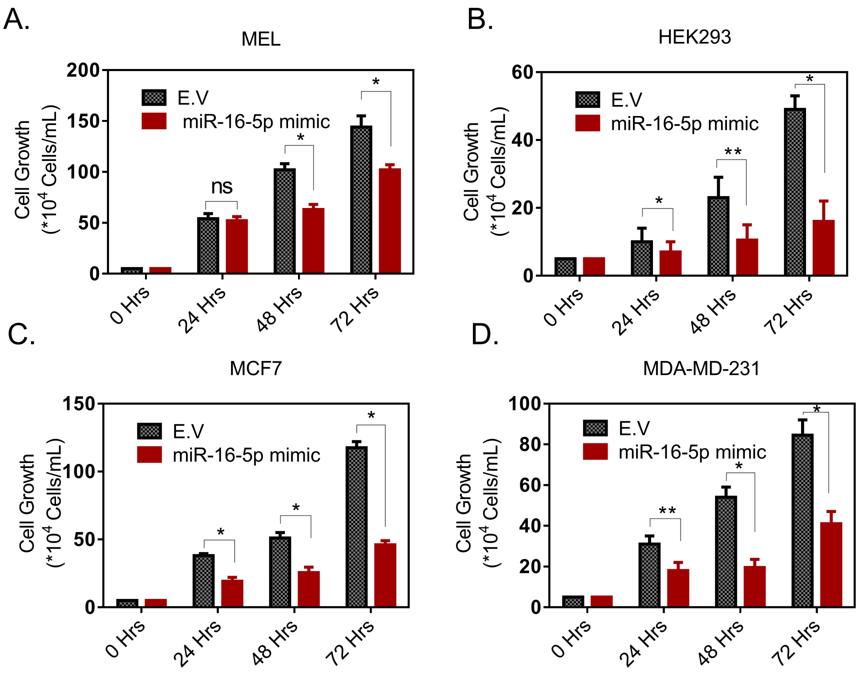

Figure S5. miR-16-5p overexpression inhibits cell proliferation of various cancer cell lines. (A-D) miR-16-5p levels were elevated by the use of miR-16-p5 mimic, as shown in Supplementary figure 3 (E-H). Cell proliferation was scored microscopically in a Neubauer chamber. Statistical significance was inferred using multiple t-tests (Sidak-Bonferroni method) between E.V. and miR-16-5p mimic treated cells. (*p<0.001, **p<0.05).

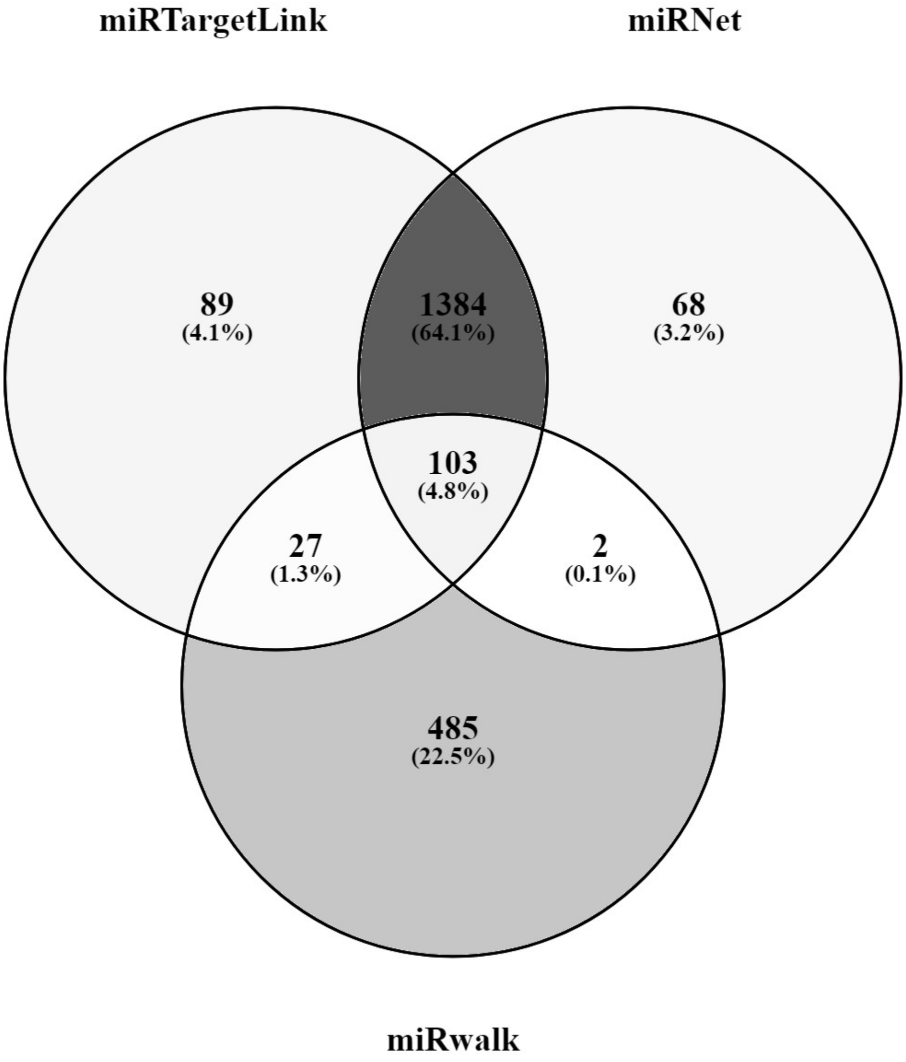

Figure S6. Venn diagram comparing the genes targeted by miR-16-5p according to three online platforms (miRTargetLink, miRNet, miRwalk). Plot was created with Venny 2.1 (Oliveros, J.C. (2007-2015) Venny. An interactive tool for comparing lists with Venn's diagrams. https://bioinfogp.cnb.csic.es/tools/venny/index.html)

Pathway	Total	Expected	Hits	Pval
Ribosome biogenesis in eukaryotes	55	5,19	19	2,59E-07
RNA transport	126	11,9	30	1,15E-06
Prostate cancer	87	8,22	22	1,14E-05
Pathways in cancer	310	29,3	52	1,83E-05
Cell cycle	124	11,7	26	6,58E-05
Small cell lung cancer	80	7,56	19	0,000114
p53 signaling pathway	68	6,42	17	0,000132
Chronic myeloid leukemia	73	6,89	17	0,000333
Insulin signaling pathway	137	12,9	26	0,000363
Neurotrophin signaling pathway	123	11,6	24	0,000397
Focal adhesion	200	18,9	34	0,000427
Acute myeloid leukemia	57	5,38	14	0,000625
Glioma	65	6,14	15	0,00082
Melanoma	68	6,42	15	0,00135
HTLV-I infection	199	18,8	32	0,00166
Oocyte meiosis	108	10,2	20	0,00232
mTOR signaling pathway	45	4,25	11	0,00246
Non-small cell lung cancer	52	4,91	12	0,00268
ErbB signaling pathway	87	8,22	17	0,00269
Wnt signaling pathway	144	13,6	24	0,00385

Table S1. The 20 most significant pathways affected by miR-16-5p retrieved through miRNET (functional enrichment tool using the embedded in miRNET tool).

Term	Count	%	P-Value	Benjamini
Ribosome	37	2,4	6,20E-08	1,70E-05
RNA transport	37	2,4	2,40E-05	2,50E-03
PI3K-Akt signaling pathway	61	3,9	2,80E-05	2,50E-03
Ribosome biogenesis in eukaryotes	23	1,5	5,00E-05	3,30E-03
Prostate cancer	23	1,5	6,10E-05	3,30E-03
Small cell lung cancer	22	1,4	1,10E-04	4,90E-03
Proteoglycans in cancer	39	2,5	1,30E-04	5,20E-03
Protein export	10	0,6	2,70E-04	9,40E-03
p53 signaling pathway	18	1,1	3,40E-04	1,00E-02
Cell cycle	26	1,7	7,50E-04	2,10E-02
Viral carcinogenesis	37	2,4	9,40E-04	2,20E-02
Signaling pathways regulating pluripotency of stem cells	28	1,8	9,60E-04	2,20E-02
Non-small cell lung cancer	15	1	1,30E-03	2,70E-02
Insulin signaling pathway	27	1,7	1,70E-03	3,30E-02
Spliceosome	26	1,7	2,10E-03	3,90E-02
Colorectal cancer	15	1	3,60E-03	6,20E-02
Acute myeloid leukemia	14	0,9	3,90E-03	6,20E-02
Protein processing in endoplasmic reticulum	30	1,9	4,10E-03	6,20E-02
Pathways in cancer	58	3,7	4,60E-03	6,70E-02

Table S2. The 20 most significant pathways affected by miR-16-5p retrieved through miRTargetlink (functional enrichment through the David bioinformatics tool).

miRNA Name	Forward Primer	miRNA Name	Forward Primer
miR-100-5p	ACCCGTAGATCCGAACTTGTG	miR-129-3p	CGGTCTGGGCTTGCAAAAA
miR-129-5p	CGGTCTGGGCTTGCAAA	U6	TGGCCCCTGCGCAAGGATG
miR-16-5p	TAGCAGCACGTAAATATTGGCG	miR-25-3p	TGCACTTGTCTCGGTCTGA
miR-19b-1-5p	CAGGTTTGCATCCAGCAAA	miR-193b-3p	GCCCACAAAGTCCCGCTAA
miR-193b-5p	GGTTTTGAGGGCGAGATGA	miR-22-3p	AAGCTGCCAGTTGAAGAACTGT
miR-1207-5p	GAGGCTGGGAGGGGAAAA	miR-501-3p	ACCCGGGCAAGGATTTGAAA
miR-663a	CCGCGGGACCGCAAAAAA	miR-29a-3p	TAGCACCATCTGAAATCGGTTA
miR-92a-3p	CACTTGTCCCGGCCTGTA	miR-19a-3p	TGTGCAAATCTATGCAAAACTGA
miR-186-5p	CAAAGAATTCTCCTTTTGGGCT	miR-652-3p	GGCGCCACTAGGGTTGTGAAA
miR-484	CAGTCCCCTCCCGATAAAA	miR-10a-5p	TACCCTGTAGATCCGAATTTGTG
miR-615-3p	AGCCTGGGTCTCCCTCTTA	miR-451a	AAACCGTTACCATTACTGAGTTAAAAAA

Gene Name	Forward Primer	Reverse Primer
Hemoglobin- β	ATGGCCTGAATCACTTGGAC	ACGATCATATTGCCCAGGAG
Hemoglobin-α1	GTCAACTTCAAGCTCCTAAGCC	GCTTAACGGTATTTGGAGGTCAG
CD71	ATTCCCTTCCTTCAATCACAC	TCTCCTTCCATATTCCCAAACAG
RPL14	GTGCCCACCAGAAGTATGTC	GTCATCTTGGCTTTCCTTTCTC
RPS6	GATTCAGCGTCTTGTTACTCC	CTCCTTAGCCTCCTTCATTCTC
RPS3	GAGCCACTTCCTTTCCTTTCAG	CCATCAGCGACAAACTTCCTC
RPLP0	ATGTTTCATTGTGGGAGCAGAC	TATGAGGCAGCAGTTTCTCCA
RPL11	TCTCCAAAGCTAGATACACTGTC	CCCAGCACCACATAGAAGTC
RPSA	CGGCGTTGTTCTGGATTCCC	CCATCTGGAAGTCAAGATTGGT

Table S3. miRNA and mRNA primers used for the qRT-PCR assays in the study.
